# Characteristic Fragmentation Behavior of Linear and Cyclic *O*-Linked Glycopeptides and Their Peptide Skeletons in MALDI-TOF/TOF MS

**DOI:** 10.3390/molecules30030711

**Published:** 2025-02-05

**Authors:** Kohki Fukushi, Shogo Urakami, Hiroshi Hinou

**Affiliations:** 1 Laboratory of Advanced Chemical Biology, Graduate School of Life Science, Hokkaido University, Sapporo 001-0021, Japan; kohki_0825@eis.hokudai.ac.jp (K.F.); urakamishogo27@eis.hokudai.ac.jp (S.U.); 2 Frontier Research Center for Advanced Material and Life Science, Faculty of Advanced Life Science, Hokkaido University, Sapporo 001-0021, Japan

**Keywords:** cyclic glycopeptide, cyclic peptide, *O*-linked glycan, threonine, antifreeze glycopeptide, MALDI-TOF MS, TOF/TOF, fragmentation, neutral loss, conformation

## Abstract

Understanding characteristic post-source decay (PSD) fragmentation patterns in tandem mass spectrometry (MS/MS) is important for the identification of target molecules. In this study, we explored the characteristic PSD patterns associated with *O*-linked glycopeptides and their cyclization using the MALDI-TOF/TOF MS analysis of linear and cyclic antifreeze glycoproteins. We performed a comparative analysis of the proton and sodium adduct ions of the peptide backbones of antifreeze glycoproteins, which have a simple repeating sequence, shedding light on the characteristics of the fragmentation of the threonine side chain and that of its cyclized form. Furthermore, the presence or absence of a glycan on the threonine side chain and its substitution with serine caused changes in its fragmentation. These findings are expected to contribute to the prediction of three-dimensional peptide structures and the search for physiologically active *O*-linked glycopeptides and cyclic (glyco)peptides.

## 1. Introduction

*O*-linked glycans represent a post-translational modification of peptides that is conserved in all organisms, and they have huge structural diversity, with each characteristic core structure’s nearby peptide linkage being highly conserved during the course of biological evolution [1]. *O*-linked glycans exist primarily in the form of glycosidic bonds to the hydroxyl groups of serine/threonine. In the animal kingdom, mucin-type glycans with α-linked N-acetylgalactosamine (GalNAc) residues on serine/threonine are involved in most life events and processes, including differentiation, growth, immunity, and symbiosis [2].

Cyclic peptides are molecules in which the peptide chains bond intramolecularly at separate positions to form a macrocyclic structure. The topological control of the conformation and functional groups associated with cyclization allows remarkable physiological activity and physical properties to be achieved with a small number of amino acids [3]. Cyclic peptide drugs, including the physiologically active cyclic peptides discovered in nature, have been widely developed and approved [4].

With the discovery of soft ionization methods, mass spectrometry has become a major technique in the exploration and structural analysis of various biomolecules [5,6]. Mass spectrometry is actively used to explore biologically active peptides and their post-translational modifications due to its rapidity, comprehensiveness, high resolution, and high sensitivity [7,8]. MS/MS fragmentation techniques are effective for peptide sequencing [9]. In the case of cyclic peptides, the molecular weight does not change after the first cleavage of the peptide bond. Different analytical methods and tools are required to understand the characteristics of cyclic peptides, as compared to those used for linear peptides [10].

Matrix-assisted laser desorption/ionization time-of-flight mass spectrometry (MALDI-TOF MS) is a soft ionization method that is widely used for the mass spectrometry of biomolecules [6]. To analyze the internal structure of a target molecule using MALDI-TOF MS, fragmentation by post-source decay (PSD) after precursor ion selection, named TOF/TOF, is mainly used [11]. In addition to the fragmentation pattern indicating the peptide sequence, in cyclic peptides, minor fragmentation becomes prominent during this PSD process. While this is ignored in linear peptides, it provides insights that are valuable in exploring the internal structures of cyclic peptides [9]. It has been reported that, in the PSD of cyclic peptides, the carbon and nitrogen involved in the peptide chain are desorbed as carbon monoxide and ammonia, respectively [10,12]. However, compared to linear peptides, there are limited reports on the fragmentation behavior of cyclic peptides. In particular, detailed studies on the fragmentation behavior of cyclic peptides with post-translational modifications, such as cyclic glycopeptides, are rare [13].

**Figure 1:**
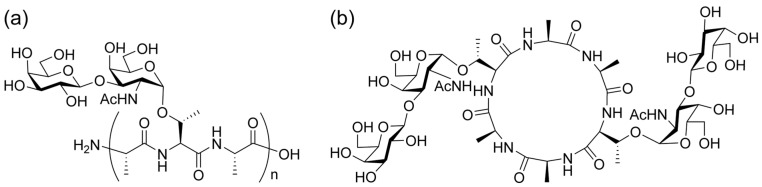
Chemical structures of (**a**) antifreeze glycoprotein (AFGP) and (**b**) cyclic AFGP (cyclized derivative of *n* = 2 AFGP).

We performed a systematic study on the synthesis and analysis of glycopeptides [14,15]. During this process, we found that cyclic glycopeptides that replicated during the synthesis of linear antifreeze glycoproteins (AFGPs; Figure 1a) in nature [16] exhibited antifreeze activity [17]. We also found that the glycan moiety of the cyclic antifreeze glycoprotein [18] (Figure 1b) could be directly observed via in-source decay (ISD) MALDI-TOF MS with a modified matrix for glycan-selective ionization [19]. The fragmentation patterns of linear *O*-linked glycopeptides have been studied and utilized in glycoproteomic studies [20,21,22,23,24]. However, to our knowledge, there have been no comparative studies with *O*-linked cyclic peptides, particularly regarding their peptide sequences and side chain fragmentation. In this study, we report the characteristic PSD patterns of linear and cyclic AFGP, as obtained via MALDI-TOF/TOF MS analysis, together with the results of a comparison with their peptide backbones.

## 2. Results and Discussion

### 2.1. Comparative MALDI-TOF/TOF MS Analysis of Linear and Cyclic AFGP

**Figure 2:**
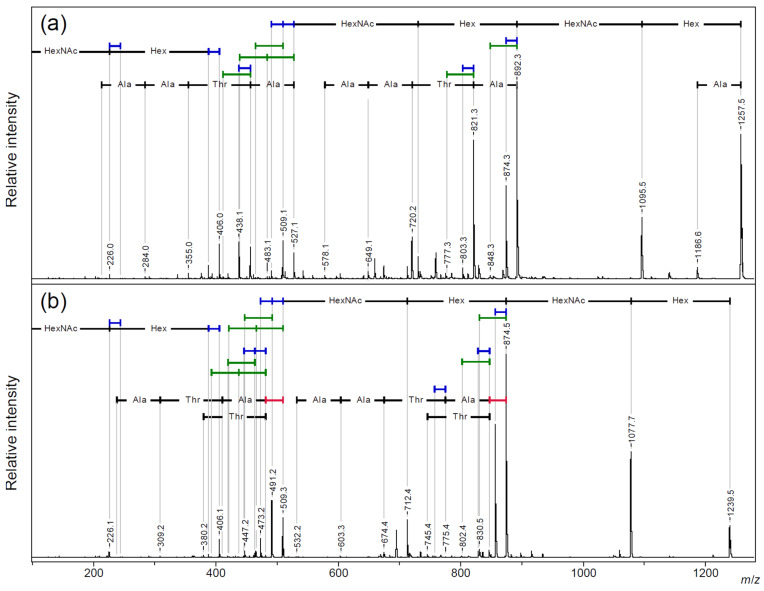
MALDI-TOF/TOF analysis of [M + Na]^+^ precursor ions for (**a**) linear (*n* = 2) and (**b**) cyclic AFGP (see Figure 1 for detailed structure). Blue bar—18 Da loss; green bar—44 Da loss; red bar—28 Da loss.

In MALDI-TOF MS, sodium adduct ions were mainly observed in both linear and cyclic AFGP [16,18]. A comparative analysis was performed using TOF/TOF on the sodium adduct precursor ions [M + Na]^+^ of the two-repeat (*n* = 2) linear and cyclic AFGP using a sodium-doped benzylhydroxylamine/2,5-dihydroxybenzoate salt (BOA/DHB/Na) matrix [25] (Figure 2 and Figure S1: *m*/*z* 600–910 and Figure S2: *m*/*z* 300–540).

In the small *m*/*z* region, the sodium adduct ions of glycan fragments were found as two pairs of 18 Da neutral loss (blue bar in figures) at *m*/*z* 226.1 and 244.1 for N-acetylgalctosamine residues (HexNAc) and *m*/*z* 388.1 and 406.1 for additional galactose residues (Hex), as reported in our ISD MALDI-TOF MS analysis of synthetic AFGP mixtures and intact mucins [19]. In the larger *m*/*z* region, the sequential neutral loss of the two pairs of disaccharide branches from the precursor ion signal gave the sodium adduct ion of the core peptide, with a free hydroxyl group of the threonine side chain and its dehydro-form as the main fragment ion signals. Following the loss of a single and a pair of disaccharide branches (*m*/*z* 892.3 and *m*/*z* 527.1, respectively), sequential neutral losses of the peptide skeleton were observed for linear AFGP (Figure 2a). Then, 28 Da neutral losses (red bar in figures) were observed before the amino acid sequence signals only in cyclic AFGP (Figure 2b). This could have been due to the loss of CO from the oxazolone structure during the ring-opening reaction, preceding the loss of amino acids [26,27,28].

Characteristic neutral loss fragment patterns of 18 Da and 44 Da (green bars in the figure) were observed for both the linear and cyclic AFGP. One neutral loss of 18 Da or 44 Da was observed for the fragment ions (*m*/*z* 892.3 and *m*/*z* 874.5, respectively) generated by the neutral loss of a disaccharide unit bound to one threonine side chain. In addition, a pair of neutral losses of 18 Da or 44 Da was observed for the fragment ions (*m*/*z* 527.1 and *m*/*z* 509.3, respectively) generated by the complete neutral loss of a pair of disaccharide side chains. These results indicate that neutral losses of 18 or 44 Da occurred from the threonine side chains.

Infrared multiphoton dissociation (IRMPD) Fourier-transform ion cyclotron resonance (FTICR) MS studies of *O*-linked glycopeptide anions reported a neutral loss of C_2_H_4_O (exact mass: 44.03) from the non-glycosylated threonine side chain [29]. However, the TOF/TOF analysis of the sodium adduct ions of AFGP showed that these neutral losses also occurred from the glycosylated threonine side chains. Wang et al. reported that PSD for the sodium adduct ion of a threonine-containing peptide resulted in a characteristic neutral loss of 44 Da, depending on the distance of the threonine residue from the C-terminal [30]. However, to our knowledge, there have been no reports on the fragmentation characteristics of threonine residues in cyclic peptides or on the neutral elimination characteristics, other than dehydration at the threonine side chain in MS/MS measurements of monoprotonated peptides observed via MALDI-TOF MS. Therefore, we performed a comparative analysis of the fragmentation patterns in the TOF/TOF analysis of the proton and sodium adduct ions of the non-glycosylated cyclic AFGP peptide backbone.

### 2.2. MALDI-TOF/TOF MS Analysis of Core Cyclic Peptide of Cyclic AFGP

**Figure 3:**
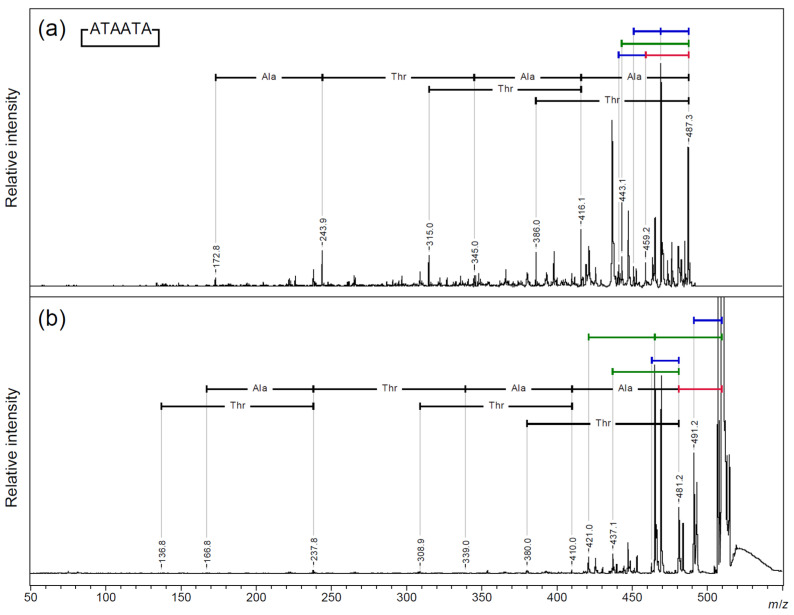
MALDI-TOF/TOF analysis of (**a**) [M + H]^+^ and (**b**) [M + Na]^+^ precursor ions of the cyclic AFGP core peptide (deglycosylated form of Figure 1b). Blue bar—18 Da loss; green bar—44 Da loss; red bar—28 Da loss.

The PSD pattern of the skeleton peptide of cyclic AFGP [31] was analyzed via MALDI-TOF/TOF MS using both proton and sodium adduct precursor ions (Figure 3 and Figure S3: *m*/*z* 300–520; Tables S3 and S4). Sequential neutral losses of amino acid residues from the proton adduct precursor ion were observed for both alanine and threonine residues (Figure 3a). From the sodium adduct ion of the cyclic peptide, sequential amino acid losses were observed after a 28 Da neutral loss from the precursor ion.

**Figure 4:**
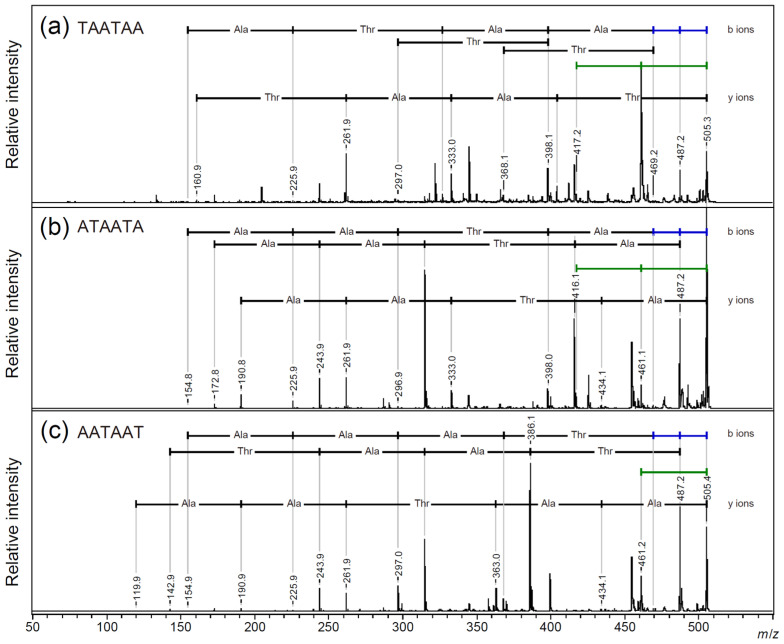
MALDI-TOF/TOF analysis of [M + H]^+^ precursor ions for the linear core peptide of AFGP: (**a**) TAATAA, (**b**) ATAATA, and (**c**) AATAAT. Blue bar—18 Da loss, green bar—44 Da loss.

**Figure 5:**
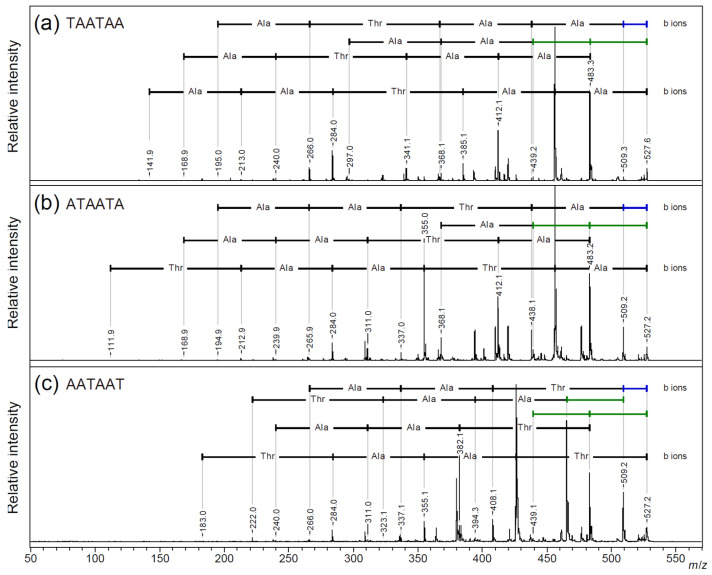
MALDI-TOF/TOF analysis of [M + Na]^+^ precursor ions for linear core peptide of AFGP: (**a**) TAATAA, (**b**) ATAATA, and (**c**) AATAAT. Blue bar—18 Da loss; green bar—44 Da loss.

The proton adduct ions produced more prominent amino acid fragment ions, but no characteristic amide bond fragmentation positions were observed in either adduct ion. Regarding the protonated ions of the cyclic peptide, the signal for the neutral loss of 18 Da was dominant and repeated twice, whereas, regarding the sodium adduct ions, the signal for the loss of 44 Da was dominant and repeated twice. In cyclic peptides, the neutral loss of an amino acid residue requires the fragmentation of two amide bonds; therefore, the fragmentation behavior of this side chain may be significantly different from that of linear peptides. Therefore, we compared the PSD fragmentation patterns of the three linear peptide skeletons of two-repeat AFGP, TAATAA, ATAATA, and AATAAT via the MALDI-TOF/TOF MS analysis of the proton and sodium adduct precursor ions (Figure 4, Figure 5 and Figure S4: *m*/*z* 315–510 and Figure S5: *m*/*z* 340–535).

Regarding the proton adduct precursor ion, only TAATAA, which had an N-terminal threonine residue, gave a 44 Da neutral loss signal as the dominant fragmentation (Figure 4a; Table S5). The proton adduct precursor ion of the ATAATA and AATAAT peptides predominantly gave an 18 Da neutral loss (Figure 4b, Table S6, Figure 4c, and Table S7). For all sequences, both b-ions and y-ions yielded the correct sequences from both terminals. Regarding the sodium adduct precursor ions of the linear peptides, all peptides gave a signal of a 44 Da neutral loss from the precursor ion, and a pair of b-ions, [b + Na]^+^ and [b + Na + OH]^+^, gave the peptide sequences [32] (Figure 5 and Figure S5; Tables S8–S10).

### 2.3. Searching for a Characteristic Fragmentation Mechanism Using Isotope-Labeled Derivatives

**Figure 6:**
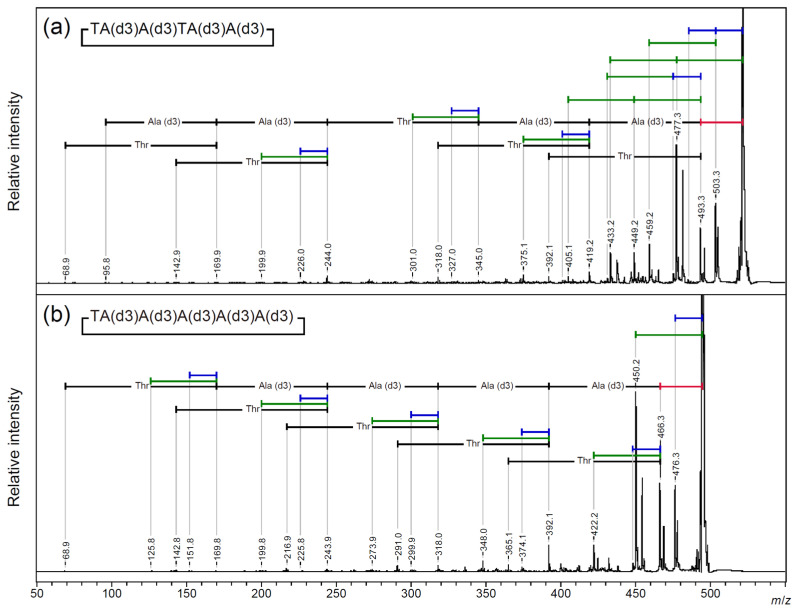
MALDI-TOF/TOF analysis of [M + Na]^+^ precursor ions for (**a**) alanine deuterium-labeled cyclic AFGP core peptide and (**b**) cyclic TA(d3)A(d3)A(d3)A(d3)A(d3). Blue bar—18 Da loss; green bar—44 Da loss; red bar—28 Da loss.

**Figure 7:**
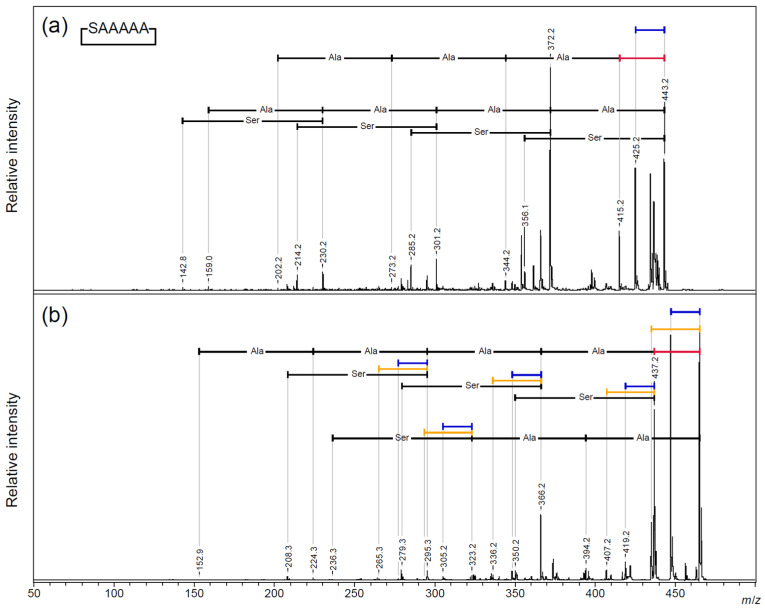
MALDI-TOF/TOF analysis of (**a**) [M + H]^+^ and (**b**) [M + Na]^+^ precursor ions for cyclic-SAAAAA. Blue bar—18 Da loss; yellow bar—30 Da loss; red bar—28 Da loss.

To clarify the origins of the 18 Da and 44 Da losses from the cyclic AFGP skeleton peptide, the deuterium-labeled alanine-3,3,3-d3 [A(d3)]-modified peptide, cyclic TA(d3)A(d3)TA(d3)A(d3), and cyclic TA(d3)A(d3)A(d3)A(d3)A(d3), one of the two threonine residues was replaced with A(d3). A MALDI-TOF/TOF MS analysis was performed on the sodium adduct precursor ions of the cyclic peptides (Figure 6 and Figure S6: *m*/*z* 330–530). From both precursor ions, a neutral loss of 44 Da was observed as the highest fragment peak. An additional neutral loss of 44 Da was observed only for the AFGP skeleton analog. The neutral loss of deuterium-labeled alanine (74 Da) was observed after a neutral loss of 28 Da. For the cyclic AFGP skeleton analog, losses of 18 Da or 44 Da were observed up to twice in total (Figure 6a; Table S11). A loss of 18 or 44 Da after a 28 Da loss was also observed. From cyclic TA(d3)A(d3)A(d3)A(d3)A(d3), a 62 Da loss corresponding to the sequential loss of 18 and 44 Da was not observed, and a simplified fragmentation pattern was observed (Figure 6b; Table S12). In addition, the MALDI-TOF/TOF MS analysis of a cyclic SAAAAA peptide, in which threonine was replaced with serine, was performed (Figure 7 and Figure S7: *m*/*z* 270–470). From protonated precursor ion, the side chain was no longer released as an aldehyde, and only a neutral loss of water was observed. In contrast to the threonine-containing cyclic peptide, at *m*/*z* 372.2, the neutral loss of an alanine residue without any other neutral loss was observed as the strongest fragment signal. Sequencing signals following a 28 Da neutral loss were also observed as minor signals compared to the direct neutral loss of amino acid sequence information (Figure 7a; Table S13). From the sodium-added precursor ion, a neutral loss of 18 Da was the strongest signal, followed by a 28 Da neutral loss, and the neutral loss of 30 Da corresponding to CH_2_O from the serine side chain was observed as a minor signal. The major sequencing signals observed were those following a 28 Da neutral loss (Figure 7b; Table S14).

### 2.4. Side Chain Elimination Mechanism and Adduct Ion Selectivity of Cyclic Peptides

**Figure 8:**
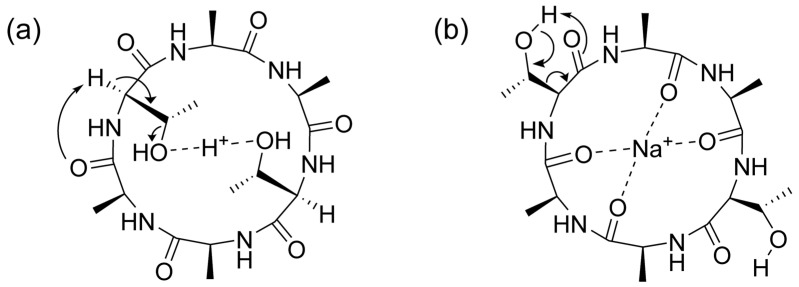
Possible mechanisms behind the neutral loss of (**a**) 18 Da from the proton adduct ion and (**b**) 44 Da from the sodium ion of the AFGP cyclic peptide skeleton.

These fragmentation patterns clearly show that both the 18 Da and 44 Da neutral losses originate from the threonine side chain. For the cyclic peptide, the protonated precursor ion was dominated by a neutral loss of 18 Da. A 44 Da neutral loss was observed as the predominant fragment signal from the sodium adduct precursor ions. These different fragmentation reactions are expected to arise from differences in the coordination sites of the adduct ions (Figure 8). The protonated ions are expected to promote dehydration by coordinating with threonine hydroxyl groups (Figure 8a). The sodium adduct ions promoted the formation of a turn structure suitable for the loss of acetaldehyde (Figure 8b). In the case of sodium adduct ions of linear and cyclic AFGP, the glycosylation of the threonine hydroxyl group inhibits the conformation suitable for de-acetaldehyde to give a 44 Da neutral loss even for the sodium adduct ion (Figure 2). The de-glycoside reaction to give an *m*/*z* 406 signal (Figure 2) as a free disaccharide also promoted the formation of a dehydrated peptide skeleton. Interestingly, the mechanism of the 44 Da neutral loss from the threonine side chain of the cyclic peptide might differ from those previously reported in [29,30] and in our study using the linear peptide skeletons of AFGP (Figure 4 and Figure 5). This is likely because threonine adopts a conformation suitable for the neutral loss of acetaldehyde due to hydrophobic interactions between the methyl groups of alanine and threonine [32]. The hydrophobic interactions of the methyl groups are an important factor in the antifreeze activity of AFGP [16]. The replacement of threonine with serine changes the conformation and antifreeze activity [16], as well as the selectivity and reactivity of the neutral loss of the side chain (Figure 6 and Figure 7). The hydroxyl groups of threonine and serine represent the main stage of post-translational modification, and the conformational changes associated with post-translational modification constitute the switch for protein function, as well as the functional control of drug candidates composed of cyclic (glyco)peptides [32,33,34].

## 3. Materials and Methods

### 3.1. Materials and Reagents

Fmoc-alanine-OH, Fmoc-threonine-OH, Fmoc-serine-OH, and 2-chloro trityl resin were purchased from Novabiochem^®^ (Merck KGaA, Darmstadt, Germany). L-Alanine-3,3,3-d3-N-Fmoc was purchased from CDN Isotopes Inc. (Pointe-Claire, QC, Canada). O-benzotriazole-N,N,N′,N′-tetramethyluronium hexafluorophosphate (HBTU), 1-hydroxybenzotriazole hydrate (HOBt·H_2_O), N,N-diisopropylethylamine (DIPEA), dichloromethane (DCM) (SP grade), dimethylformamide (DMF) (SP grade), methanol, piperidine, sodium hydroxide, 2,5-dihydroxybenzoic acid (DHB), sodium bicarbonate (NaHCO_3_), O-benzylhydroxylamine (BOA), acetonitrile (HPLC grade), and diisopropylcarbodiimide (DIC) were purchased from the FUJIFILM Wako Pure Chemical Corporation (Osaka, Japan). Hydroxybenzotriazole active ester (HOAt) was purchased from the GenScript Biotech Corporation (Piscataway, NJ, USA). Hexafluoroisopropanol (HFIP), 2,2,2-trifluoroethanol (TFE), and thionyl chloride (SOCl_2_) were purchased from Tokyo Chemical Industry Co., Ltd. (Tokyo, Japan). Trityl-OH ChemMatrix resin was purchased from Biotage AB (Uppsala, Sweden). Additionally, 700 µm STA µFocus plates measuring 24 × 16 c were purchased from Hudson Surface Technology (Fort Lee, NJ, USA).

### 3.2. Synthesis and Cyclization of Linear AFGP (n = 2)—Related Peptides

The Trityl-OH ChemMatrix resin was chlorinated through treatment with 2% SOCl_2_ in DCM, and the reaction mixture was stirred overnight with four equivalents of the first amino acid and six equivalents of DIEA. After the completion of the reaction, MeOH was added to quench any unreacted sites, and the resin was washed three times with DMF, DCM, and DMF. Fmoc deprotection was performed using 20% piperidine in DMF under microwave irradiation (0–40 W) at 50 °C for 3 min. The coupling reaction was carried out by adding 4 equivalents of the amino acid (or 1.5 equivalents of glyco-amino acids), 4 equivalents of HBTU, HOBt, and 6 equivalents of DIEA under microwave irradiation (0–40 W) at 50 °C for 9 min. For glyco-amino acids, two equivalents of HBTU and HOBt and four equivalents of DIEA were added to the reaction. The resin was cleaved via stirring in HFIP/DCM (1:4 *v*/*v*) for 2 h, followed by freeze-drying. The resulting product was then subjected to deacetylation via treatment with a NaOH/MeOH solution adjusted to pH 12.5 [35]. The products were analyzed after deacetylation.

The synthesis of cyclic AFGP was carried out similarly to that of the naked peptide version [31]. After cleavage, the sample was dissolved in TFE/DCM (1:1 *v*/*v*) solution at 1 mM. Five equivalents of HOAt and DIC were added. The mixture was stirred overnight at room temperature and dried, and the sugars were deacetylated before analysis.

### 3.3. Synthesis of Linear Peptides and Their Cyclization

A 2-chrolo trityl resin was used for peptides without glycan. The resin was swollen in DCM, and four equivalents of Fmoc amino acid and six equivalents of DIEA were added. The mixture was then stirred overnight at room temperature. After the incorporation of the first residue, MeOH was added, and the resin was washed three times with DMF, DCM, and DMF. Fmoc deprotection was performed using 20% piperidine in DMF.

The coupling reaction was performed by adding four equivalents of the amino acid, four equivalents of HBTU, HOBt, and six equivalents of DIEA under microwave irradiation (0–40 W) at 50 °C for 9 min. The amino acids were then incorporated into the resin. Six linear peptides were synthesized using this method: H-TAATAA-OH, H-ATAATA-OH, H-AATAAT-OH, H-TA(d3)A(d3)TA(d3)A(d3)-OH, H-TA(d3)A(d3)A(d3)A(d3)A(d3)-OH, and H-SAAAAA-OH.

The first three peptides (H-TAATAA-OH, H-ATAATA-OH, and H-AATAAT-OH) were analyzed directly, without further modification. H-TAATAA-OH was also subjected to cyclization, along with the remaining three peptides (H-TA(d3)A(d3)TA(d3)A(d3)-OH, H-TA(d3)A(d3)A(d3)A(d3)A(d3)-OH, and H-SAAAAA-OH), using the same method as for cyclic AFGP synthesis. Resin cleavage was performed by stirring the resin in HFIP/DCM (1:4 *v*/*v*) for 2 h. After cleavage, the peptides were dissolved in a (1:1 *v*/*v*) TFE/DCM solution at a concentration of 1 mM. HOAt and DIC (five equivalents) were added to this solution, and the mixture was stirred overnight at room temperature. The products were then freeze-dried and analyzed.

### 3.4. MALDI-TOF/TOF Analysis

The matrix used for the proton adduct ion analysis was prepared using DHB (10 mM) in a water/acetonitrile/TFA mixture (50:50:0.1, *v*/*v*/*v*). For the sodium adduct ion analysis, the matrix consisted of BOA/DHB/Na (12 mM/10 mM/1 mM) in a water/acetonitrile mixture (1:1, *v*/*v*) [25]. The samples were dissolved in a water/acetonitrile mixture (1:1, *v*/*v*) at a concentration of 1 μg/μL. A 0.35 μL aliquot of the sample solution was mixed with 0.35 μL of the matrix solution, spotted onto a MALDI plate (STA μFocus plate, 24 × 16, 700 μm), and air-dried before measurement.

All tandem mass spectrometry (MS/MS) analyses were performed using an Ultraflex III MALDI-TOF/TOF instrument (Bruker, Bremen, Germany) equipped with a 200 Hz Smartbeam Nd:YAG laser (355 nm).

For the TOF/TOF analysis [36], the precursor ions were initially accelerated to 8.0 kV in the MALDI ion source and selected within a time gate. The selected ions were further accelerated to 19.0 kV in the LIFT cell for fragmentation, and metastable post-source decay (PSD) ions were analyzed without additional fragmentation, such as collision-induced dissociation.

The two types of AFGP were analyzed using 1200 laser shots in parent mode (40% laser power) and 1200 laser shots in fragment mode (72% laser power). Similarly, the four types of cyclic peptides were analyzed with 400 laser shots in parent mode (40% laser power) and 800 laser shots in fragment mode (72% laser power).

## 4. Conclusions

In this study, we identified characteristic small-molecule neutral loss patterns using the MALDI-TOF/TOF MS of linear and cyclic antifreeze glycoproteins and their skeleton peptides. These neutral losses occurred due to the elimination of carbon monoxide (28 Da) associated with ring opening, the dehydration (18 Da) of the C-terminal, and the competitive elimination of water (18 Da) and acetaldehyde (44 Da) from the threonine side chain. During the competitive elimination of the threonine side chain, the ratio changed significantly depending on the difference in the adduct ion, the presence or absence of a glycosylated threonine residue, and substitution with serine.

The selectivity of this neutral loss depends on the position of the hydrogen bond and the conformational changes in the threonine side chain. This study proposes a new fragmentation mechanism for cyclic peptides, derived using mass spectrometry, and shows the possibility of using this mechanism for conformational analysis. Applied research, such as the search for physiologically active cyclic (glyco)peptides and functional prediction, is expected.

## Data Availability

All data are available upon request.

## Supplementary Materials

The following supporting information can be downloaded at: https://www.mdpi.com/article/10.3390/molecules30030711/s1. Figure S1: Enlarged view of Figure 2 (*m*/*z* 600–910); Figure S2: Enlarged view of Figure 2 (*m*/*z* 300–540); Figure S3: Enlarged view of Figure 3 (*m*/*z* 300–520); Figure S4: Enlarged view of Figure 4 (*m*/*z* 315–510); Figure S5: Enlarged view of Figure 5 (*m*/*z* 340–535); Figure S6: Enlarged view of Figure 6 (*m*/*z* 330–530); Figure S7: Enlarged view of Figure 7 (*m*/*z* 270–470); Table S1: Mass list of fragment ion peaks from linear AFGP (*n* = 2, precursor ion *m*/*z* 1257.5) in Figure 2a; Table S2: Mass list of fragment ion peaks from cyclic AFGP (precursor ion *m*/*z* 1239.5) in Figure 2b; Table S3: Mass list of fragment ion peaks from proton adduct ion of cyclic AFGP core peptide (precursor ion *m*/*z* 487.2) in Figure 3a; Table S4: Mass list of fragment ion peaks from sodium adduct ion of cyclic AFGP core peptide (precursor ion *m*/*z* 509.2) in Figure 3b; Table S5: Mass list of fragment ion peaks from proton adduct ion of linear TAATAA (precursor ion *m*/*z* 505.2) in Figure 4a; Table S6: Mass list of fragment ion peaks from proton adduct ion of linear ATAATA (precursor ion *m*/*z* 505.2) in Figure 4b; Table S7: Mass list of fragment ion peaks from proton adduct ion of linear AATAAT (precursor ion *m*/*z* 505.2) in Figure 4c; Table S8: Mass list of fragment ion peaks from sodium adduct ion of linear TAATAA (precursor ion *m*/*z* 527.2) in Figure 5a; Table S9: Mass list of fragment ion peaks from sodium adduct ion of linear ATAATA (precursor ion *m*/*z* 527.2) in Figure 5b; Table S10: Mass list of fragment ion peaks from sodium adduct ion of linear AATAAT (precursor ion *m*/*z* 527.2) in Figure 5c; Table S11: Mass list of fragment ion peaks from sodium adduct ion of alanine deuterium-labeled cyclic AFGP core peptide (precursor ion *m*/*z* 521.2) in Figure 6a; Table S12: Mass list of fragment ion peaks from sodium adduct ion of cyclic TA(d3)A(d3)A(d3)A(d3)A(d3) (precursor ion *m*/*z* 494.2) in Figure 6b; Table S13: Mass list of fragment ion peaks from proton adduct ion of cyclic SAAAAA (precursor ion *m*/*z* 443.2) in Figure 7a; Table S14: Mass list of fragment ion peaks from proton adduct ion of cyclic SAAAAA (precursor ion *m*/*z* 465.2) in Figure 7b.
