# Supplementary material for: Characteristic Fragmentation Behavior of Linear and Cyclic O-Linked Glycopeptides and Their Peptide Skeletons in MALDI-TOF/TOF MS
Source: Molecules. 2025 Feb 5;30(3):711. doi: 10.3390/molecules30030711 (PMC11821001; doi:10.3390/molecules30030711)
Supplement: Supplementary file 1 [file molecules-30-00711-s001.zip › molecules-3430779-supplementary.pdf]

Supplementary Materials

Characteristic Fragmentation Behavior of Linear and Cyclic *O*-Linked Glycopeptides and Their Peptide Skeletons in MALDI-TOF/TOF MS

Kohki Fukushi, Shogo Urakami, and Hiroshi Hinou*

Contents

Figure S1~S7.

Table S1~S14

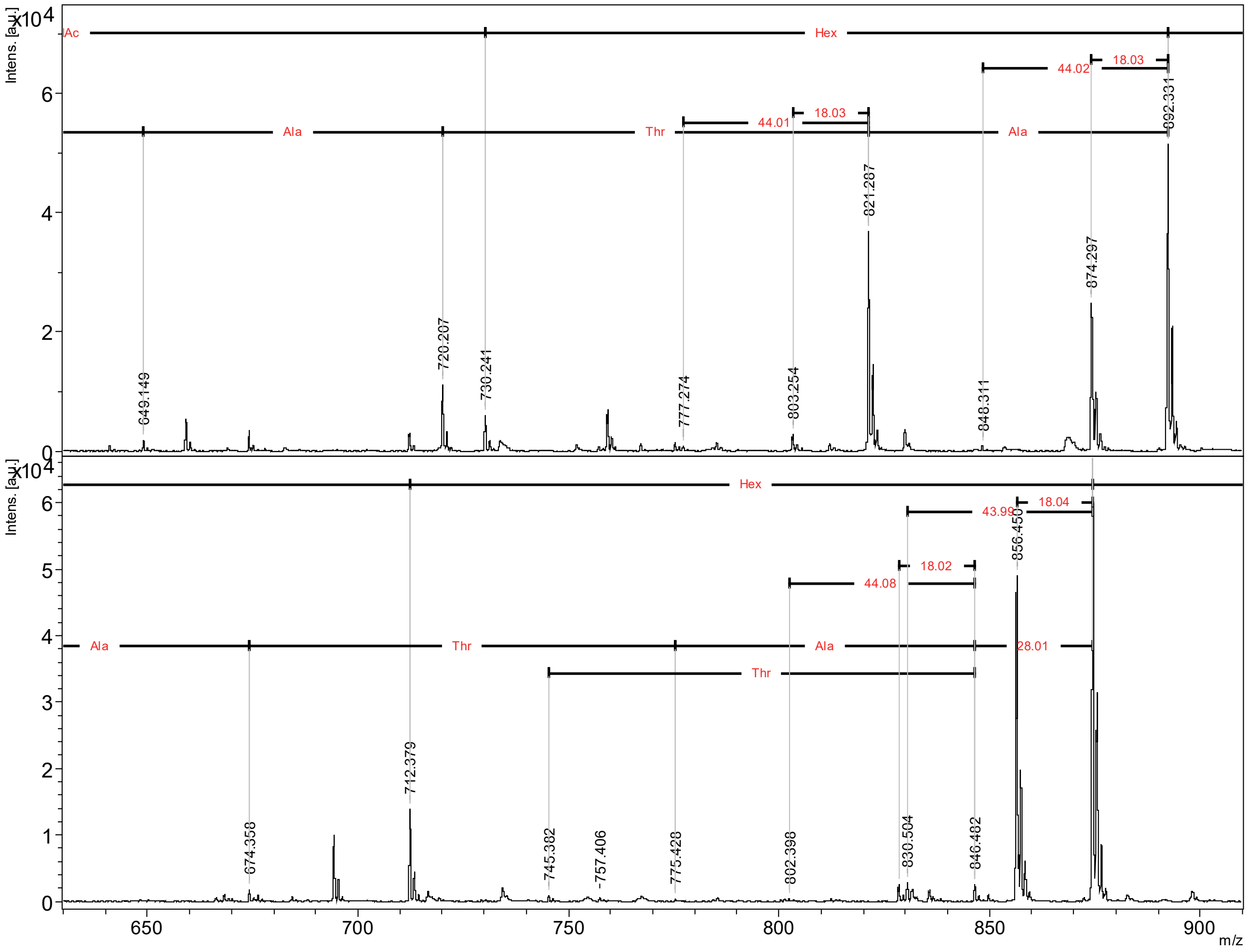

Figure S1. Enlarged view of Figure 2 (*m/z* 630-910).

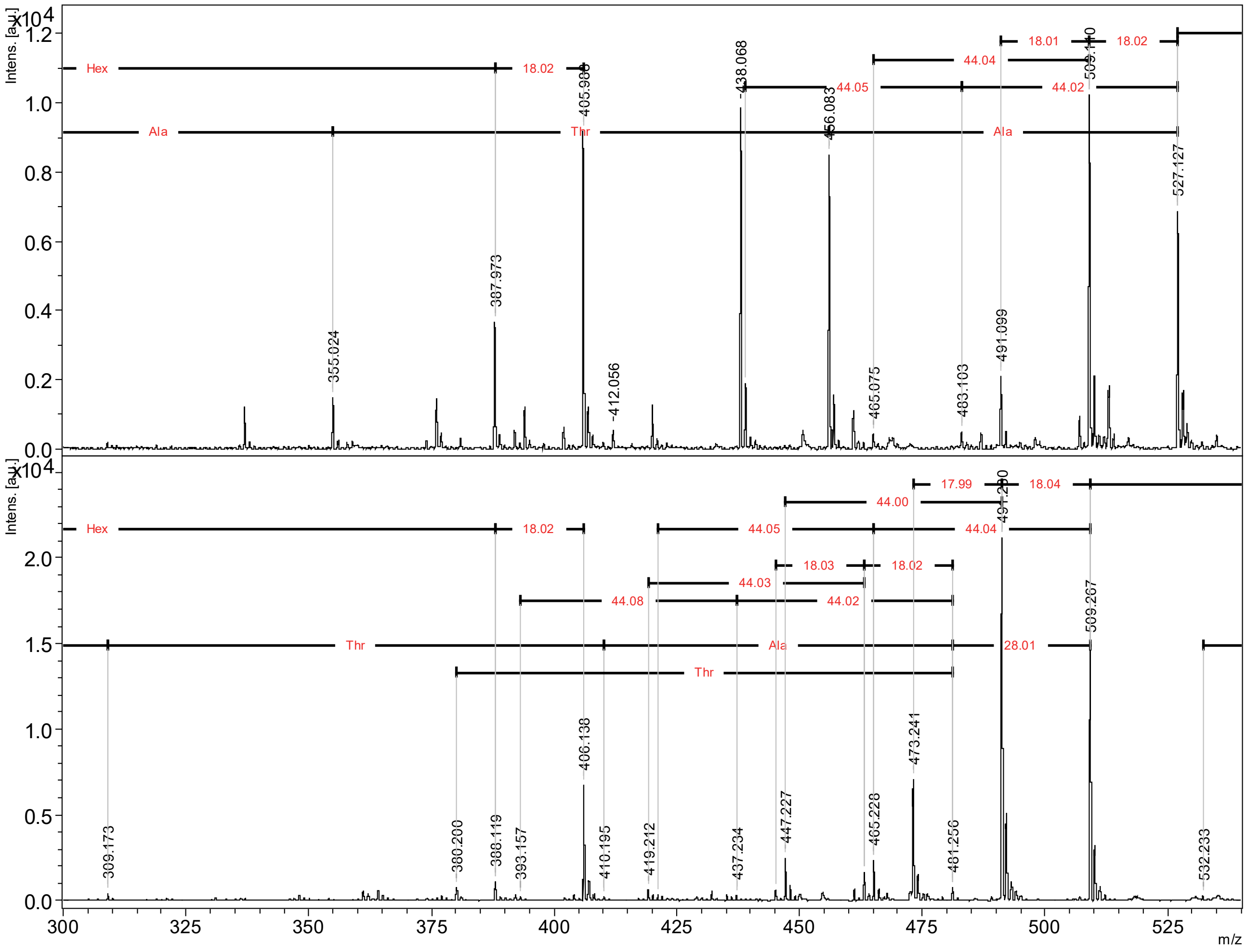

Figure S2. Enlarged view of Figure 2 (*m*/*z* 300-540).

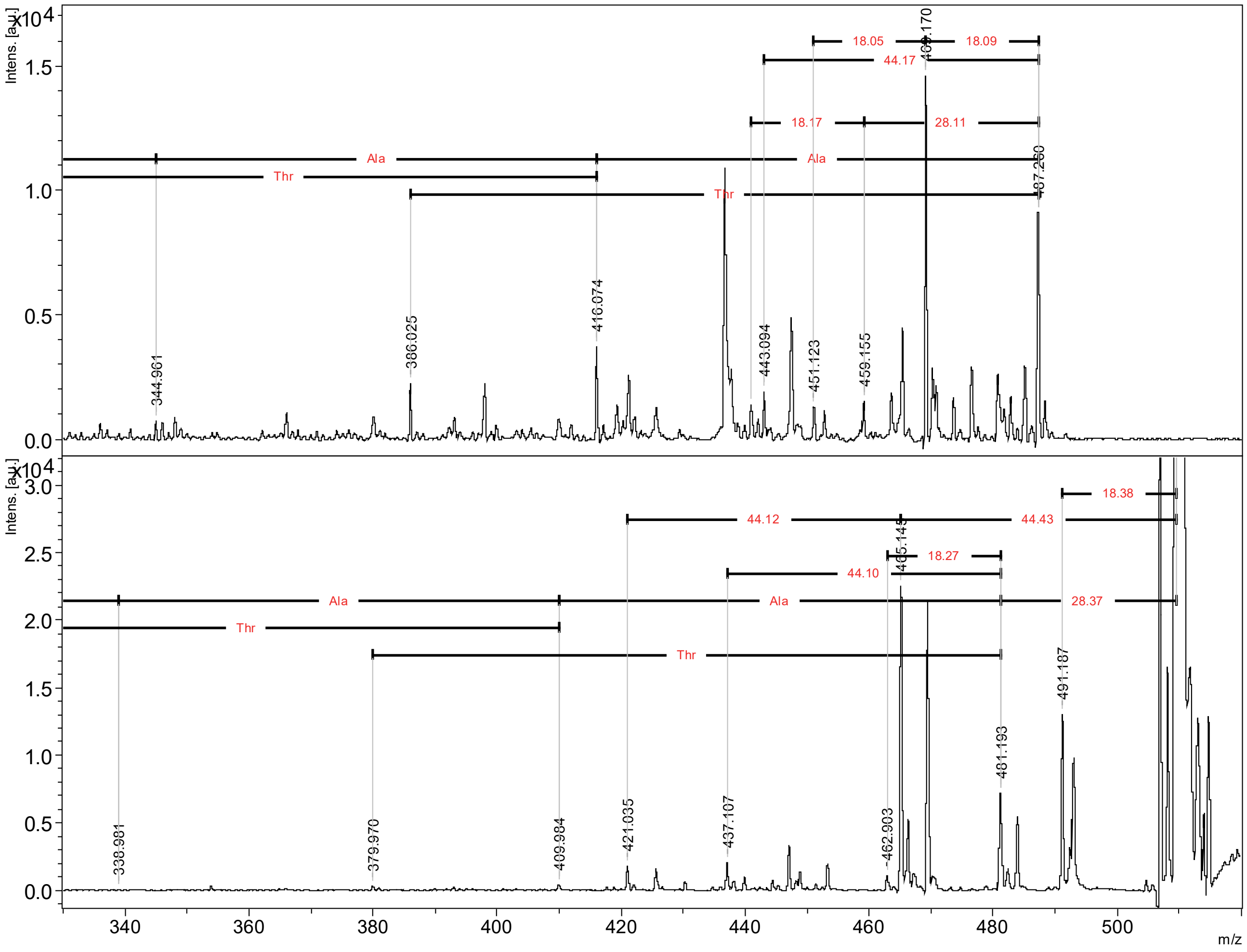

Figure S3. Enlarged view of Figure 3 (*m/z* 330-520).

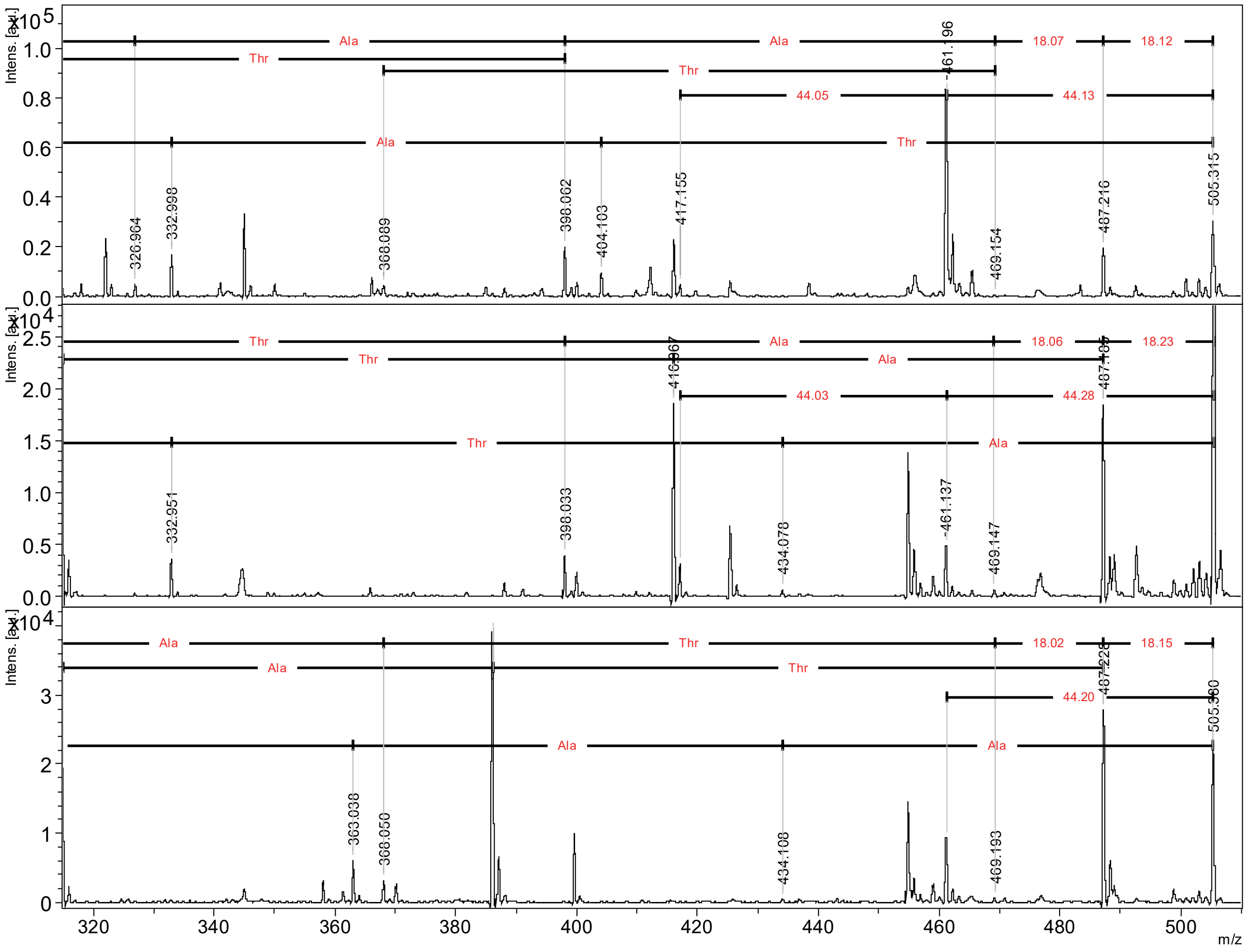

Figure S4. Enlarged view of Figure 4 (*m*/*z* 315-510).

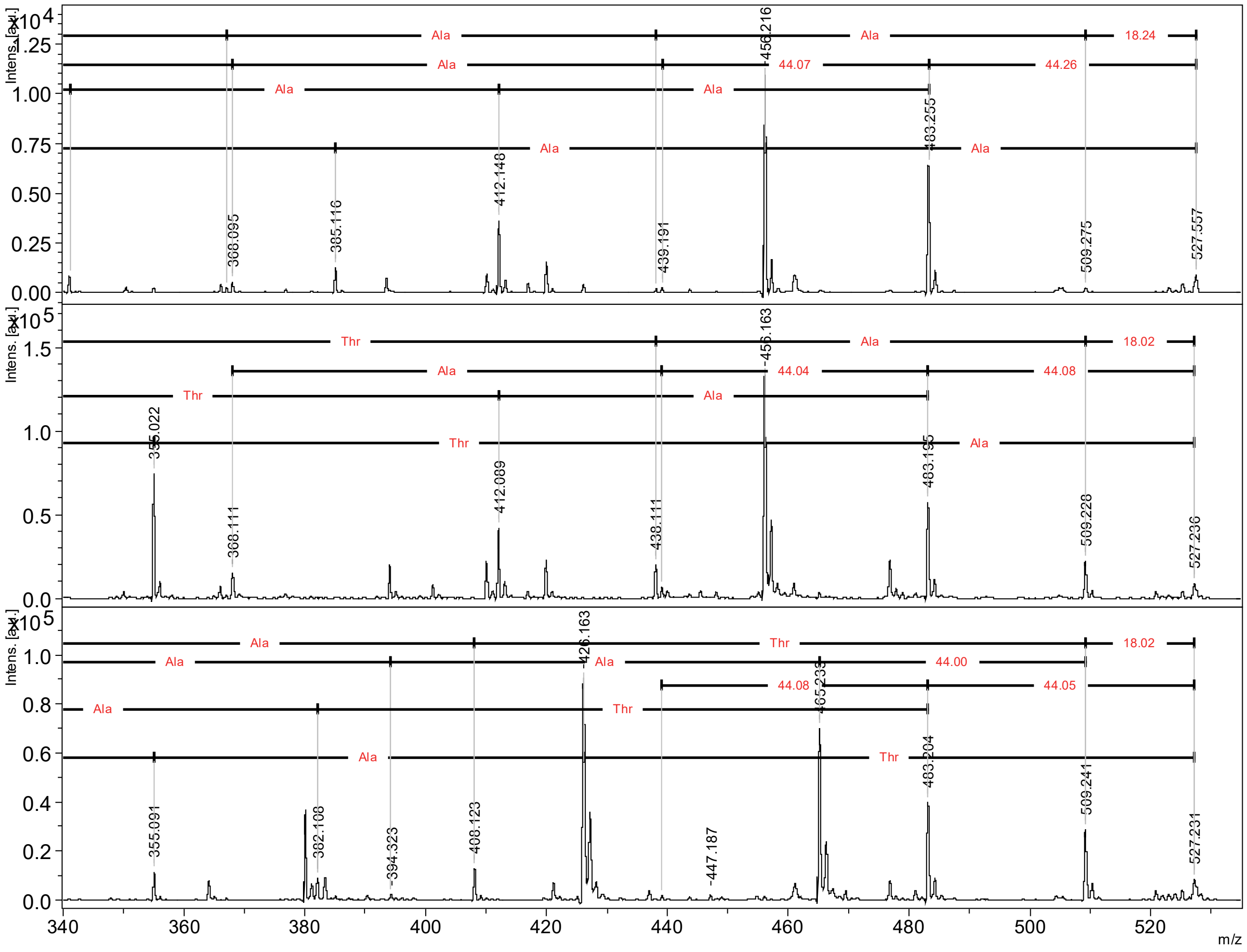

Figure S5. Enlarged view of Figure 5 (m/z 340-535).

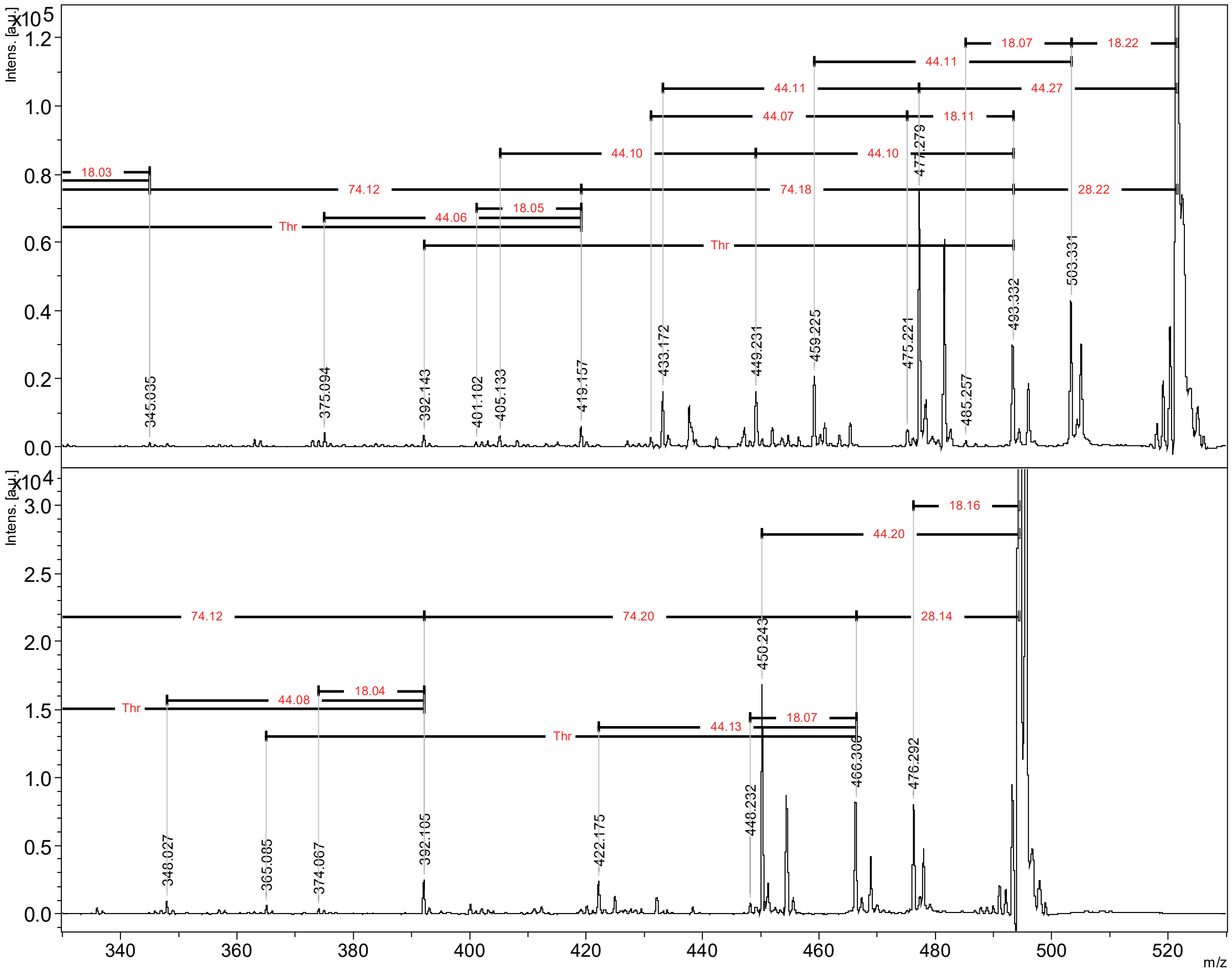

Figure S6. Enlarged view of Figure 6 (*m/z* 330-530).

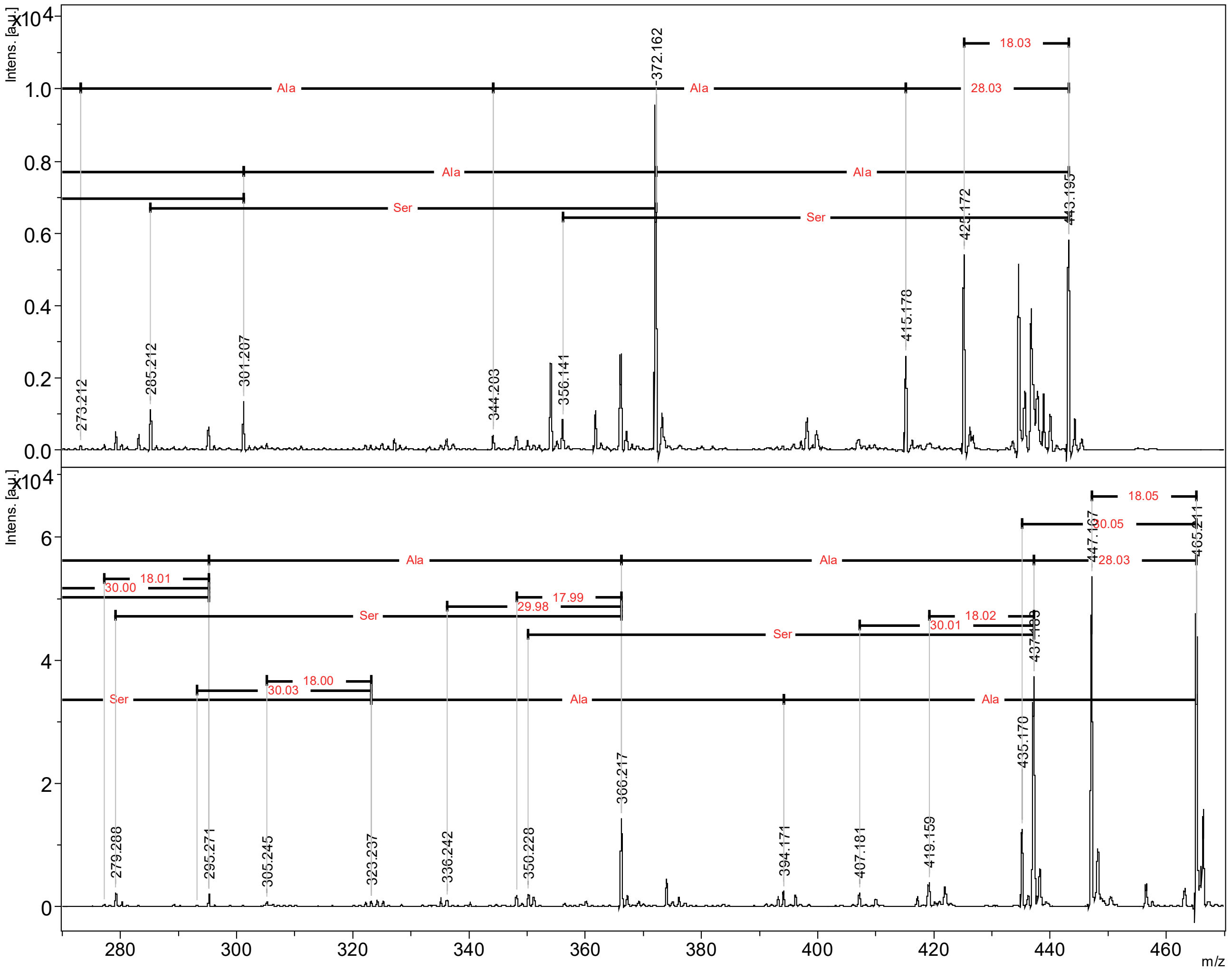

Figure S7. Enlarged view of Figure 7 (*m*/*z* 270-470).

Table S1. Mass list of fragment ion peaks from linear AFGP (n = 2, precursor ion *m*/*z* 1257.5) in Figure 2a.

Structure	Calculated *m*/*z*	Detec ted *m*/*z*	Δ*m*/*z*	Intensity	S/N
$[M + Na]^+$	1257.509	1257.495	0.015	38468.994	736.101
$[M - Ala + Na]^+$	1186.472	1186.558	0.088	3092.361	50.890
$[M - Hex + Na]^+$	1095.456	1095.521	0.061	16410.999	221.051
$[M - Hex - HexNAc + Na]^+$	892.377	892.331	0.049	51567.011	329.401
$[M - Hex - HexNAc - H_2O + Na]^+$	874.366	874.297	0.073	24841.012	148.462
$[M - Hex - HexNAc - C_2H_4O + Na]^+$	848.350	848.311	0.039	846.019	4.883
$[M - Hex - HexNAc - Ala + Na]^+$	821.339	821.287	0.053	36891.250	194.996
$[M - Hex - HexNAc - Ala - H_2O + Na]^+$	803.329	803.254	0.076	2827.698	14.324
$[M - Hex - HexNAc - Ala - C_2H_4O + Na]^+$	777.313	777.274	0.036	832.784	4.197
$[M - 2Hex - HexNAc + Na]^+$	730.324	730.241	0.079	6027.434	32.948
$[M - Hex - HexNAc - Ala -Thr + Na]^+$	720.292	720.207	0.083	11175.994	61.092
$[M - Hex - HexNAc - 2Ala -Thr + Na]^+$	649.255	649.149	0.101	1870.557	13.277
$[M - Hex - HexNAc - 3Ala -Thr + Na]^+$	578.218	578.078	0.142	1003.796	9.655
$[M - 2Hex - 2HexNAc + Na]^+$	527.244	527.127	0.113	6880.405	73.494
$[M - 2Hex - 2HexNAc - H_2O + Na]^+$	509.234	509.110	0.120	10257.390	111.363
$[M - 2Hex - 2HexNAc - 2H_2O + Na]^+$	491.223	491.099	0.121	2116.764	23.142
$[M - 2Hex - 2HexNAc - C_2H_4O + Na]^+$	483.218	483.103	0.117	496.762	5.672
$[M - 2Hex - 2HexNAc - H_2O - C_2H_4O + Na]^+$	465.208	465.075	0.135	443.242	5.217
$[M - 2Hex - 2HexNAc - Ala + Na]^+$	456.210	456.083	0.127	8513.855	101.935
$[M - 2Hex - 2HexNAc - 2C_2H_4O + Na]^+$	439.192	439.053	0.137	1886.133	23.026
$[M - 2Hex - 2HexNAc - Ala - H_2O + Na]^+$	438.197	438.068	0.132	9870.855	120.504
$[M - 2Hex - 2HexNAc - Ala - C_2H_4O + Na]^+$	412.181	412.056	0.124	548.589	7.334
$[M - 2Hex - 2HexNAc - Ala - Thr + Na]^+$	355.160	355.024	0.136	1484.429	27.558
$[M - 2Hex - 2HexNAc - 2Ala - Thr + Na]^+$	284.122	284.008	0.112	541.670	14.046
$[M - 2Hex - 2HexNAc - 3Ala - Thr + Na]^+$	213.085	212.948	0.142	77.309	2.236
$[Hex + HexNAC + H_2O + Na]^+$	406.132	405.988	0.144	9199.658	130.971
$[Hex + HexNAC + Na]^+$	388.121	387.973	0.148	3687.994	54.685
$[HexNAc + H_2O + Na]^+$	244.079	243.974	0.105	234.104	6.583
$[HexNAc + Na]^+$	226.069	225.957	0.112	1070.453	30.712

Table S2. Mass list of fragment ion peaks from cyclic AFGP (precursor ion *m/z* 1239.5) in Figure 2b.

Structure	Calculated *m/z*	Detected *m/z*	Δ*m/z*	Intensity	S/N
$[M + Na]^+$	1239.498	1239.490	0.008	10882.047	284.459
$[M - Hex + Na]^+$	1077.445	1077.668	0.223	39036.855	668.010
$[M - Hex - HexNAc + Na]^+$	874.366	874.495	0.129	73990.792	592.338
$[M - Hex - HexNAc - H_2O + Na]^+$	856.355	856.450	0.095	47989.665	374.108
$[M - Hex - HexNAc - CO + Na]^+$	846.370	846.482	0.112	2509.980	19.154
$[M - Hex - HexNAc - C_2H_4O + Na]^+$	830.340	830.504	0.164	2841.836	20.975
$[M - Hex - HexNAc - CO - H_2O + Na]^+$	828.361	828.467	0.106	2458.906	18.017
$[M - Hex - HexNAc - CO - C_2H_4O + Na]^+$	802.345	802.398	0.053	423.473	3.113
$[M - Hex - HexNAc - CO - Ala + Na]^+$	775.334	775.428	0.094	435.025	3.131
$[M - Hex - HexNAc - CO - Ala - H_2O + Na]^+$	757.323	757.406	0.083	630.347	4.679
$[M - Hex - HexNAc - CO - Thr + Na]^+$	745.323	745.382	0.059	1080.349	8.150
$[M - 2Hex - HexNAc + Na]^+$	712.313	712.379	0.066	13818.272	116.426
$[M - Hex - HexNAc - CO - Ala - Thr + Na]^+$	674.286	674.358	0.072	1908.210	18.559
$[M - Hex - HexNAc - CO - 2Ala - Thr + Na]^+$	603.249	603.294	0.045	499.653	5.928
$[M - Hex - HexNAc - CO - 3Ala - Thr + Na]^+$	532.212	532.233	0.021	241.491	3.188
$[M - 2Hex - 2HexNAc + Na]^+$	509.234	509.267	0.033	14824.781	196.592
$[M - 2Hex - 2HexNAc - H_2O + Na]^+$	491.223	491.230	0.007	21027.532	285.946
$[M - 2Hex - 2HexNAc - CO + Na]^+$	481.239	481.256	0.017	803.009	10.999
$[M - 2Hex - 2HexNAc - 2H_2O + Na]^+$	473.213	473.241	0.028	7107.503	98.074
$[M - 2Hex - 2HexNAc - C_2H_4O + Na]^+$	465.208	465.228	0.020	2384.128	32.771
$[M - 2Hex - 2HexNAc - CO - H_2O + Na]^+$	463.228	463.238	0.010	1696.016	23.312
$[M - 2Hex - 2HexNAc - C_2H_4O - H_2O+ Na]^+$	447.197	447.227	0.030	2583.397	37.099
$[M - 2Hex - 2HexNAc - CO - 2H_2O + Na]^+$	445.218	445.212	0.006	648.318	9.017
$[M - 2Hex - 2HexNAc - CO - C_2H_4O + Na]^+$	437.213	437.234	0.021	279.149	4.023
$[M - 2Hex - 2HexNAc - 2C_2H_4O + Na]^+$	421.181	421.180	0.001	337.758	4.896
$[M - 2Hex - 2HexNAc - CO - C_2H_4O - H_2O + Na]^+$	419.202	419.212	0.010	641.424	9.297
$[M - 2Hex - 2HexNAc - CO - Ala + Na]^+$	410.202	410.195	0.007	212.314	3.434
$[M - 2Hex - 2HexNAc - CO - 2C_2H_4O + Na]^+$	393.186	393.157	0.029	199.813	3.141
$[M - 2Hex - 2HexNAc - CO - Ala - H_2O + Na]^+$	392.191	392.187	0.004	381.913	6.004
$[M - 2Hex - 2HexNAc - CO - Thr + Na]^+$	380.191	380.200	0.009	791.729	14.427
$[M - 2Hex - 2HexNAc - CO - Ala - C_2H_4O + Na]^+$	366.176	366.174	0.002	175.041	3.270
$[M - 2Hex - 2HexNAc - CO - Ala - Thr + Na]^+$	309.154	309.173	0.019	457.165	11.418
$[M - 2Hex - 2HexNAc - CO - 2Ala - Thr + Na]^+$	238.117	238.114	0.003	157.353	4.382
$[Hex + HexNAC + H_2O + Na]^+$	406.132	406.138	0.006	6782.601	103.570

[Hex + HexNAC + Na]$^+$	388.121	388.119	0.002	1100.090	18.914
[HexNAc + H_2O + Na]$^+$	244.079	244.102	0.023	327.082	9.021
[HexNAc + Na]$^+$	226.069	226.090	0.021	2200.681	61.004

Table S3. Mass list of fragment ion peaks from proton adduct ion of cyclic AFGP core peptide (precursor ion *m/z* 487.2) in Figure 3a.

Structure	Calculated *m/z*	Detected *m/z*	Δ*m/z*	Intensity	S/N
$[M + H]^+$	487.251	487.260	0.009	9123.686	62.883
$[M - H_2O + H]^+$	469.240	469.170	0.070	14618.193	85.175
$[M - CO + H]^+$	459.256	459.155	0.101	1533.527	8.227
$[M - 2H_2O + H]^+$	451.230	451.123	0.107	1297.877	6.294
$[M - C_2H_4O + H]^+$	443.225	443.094	0.131	1902.719	8.636
$[M - CO - H_2O + H]^+$	441.245	440.987	0.258	1383.258	6.278
[M - Ala + H]$^+$	416.214	416.074	0.140	3718.008	15.830
[M - Thr + H]$^+$	386.203	386.025	0.178	2241.093	11.495
[M - 2Ala + H]$^+$	345.177	344.961	0.216	715.194	4.269
[M - Ala -Thr + H]$^+$	315.166	314.928	0.238	2014.554	15.167
[M - 2Ala -Thr + H]$^+$	244.129	243.875	0.254	2337.811	45.247
[M - 3Ala -Thr + H]$^+$	173.092	172.833	0.259	561.437	33.993

Table S4. Mass list of fragment ion peaks from sodium adduct ion of cyclic AFGP core peptide (precursor ion *m*/*z* 509.2) in Figure 3b.

Structure	Calculated *m*/*z*	Detected *m*/*z*	Δ*m*/*z*	Intensity	S/N
$[M + Na]^+$	509.234	509.537	0.303	153848.445	327.403
$[M - H_2O + Na]^+$	491.223	491.187	0.036	13027.097	23.473
$[M - CO + Na]^+$	481.239	481.193	0.046	7163.591	12.007
$[M - C_2H_4O + Na]^+$	465.208	465.145	0.063	22574.253	40.568
$[M - CO - H_2O + Na]^+$	463.228	462.903	0.325	1122.708	2.138
$[M - CO - C_2H_4O + Na]^+$	437.213	437.107	0.106	2072.596	11.733
$[M - 2C_2H_4O + Na]^+$	421.181	421.035	0.146	1769.090	12.249
$[M - CO - Ala + Na]^+$	410.202	409.984	0.218	406.008	3.325
$[M - CO - Thr + Na]^+$	380.191	379.970	0.221	326.203	3.699
$[M - CO - 2Ala + Na]^+$	339.165	338.981	0.184	54.261	1.427
$[M - CO - Ala - Thr + Na]^+$	309.154	308.873	0.281	226.034	9.721
$[M - CO - 2Ala - Thr + Na]^+$	238.117	237.833	0.284	300.225	29.899
$[M - CO - 3Ala - Thr + Na]^+$	167.080	166.779	0.301	26.872	2.677
$[M - CO - 2Ala - 2Thr + Na]^+$	137.069	136.803	0.266	27.829	2.614

Table S5. Mass list of fragment ion peaks from proton adduct ion of linear TAATAA (precursor ion *m*/*z* 505.2) in Figure 4a.

Structure	Calculated *m*/*z*	Detected *m*/*z*	Δ*m*/*z*	Intensity	S/N
$[M + H]^+$	505.263	505.315	0.052	30744.249	106.031
$[M - H_2O + H]^+$	487.253	487.216	0.037	19547.406	55.571
$[M - 2H_2O + H]^+$	469.242	469.154	0.088	647.522	1.432
$[M - C_2H_4O + H]^+$	461.297	461.196	0.101	83922.616	185.570
$[M - 2C_2H_4O + H]^+$	417.211	417.155	0.056	4916.403	7.392
$[M - Thr + H]^+$	404.216	404.103	0.113	9658.438	13.991
$[M - 2H_2O - Ala + H]^+$	398.205	398.062	0.143	20357.531	29.528
$[M - 2H_2O - Thr + H]^+$	368.195	368.089	0.106	4296.325	5.927
$[M - Ala - Thr + H]^+$	333.179	332.998	0.181	16978.513	27.888
$[M - 2H_2O - 2Ala + H]^+$	327.168	326.964	0.204	4787.948	8.242
$[M - 2H_2O - Ala - Thr + H]^+$	297.157	296.953	0.204	1432.816	3.178
$[M - 2Ala - Thr + H]^+$	262.141	261.928	0.213	29395.253	97.220
$[M - 2H_2O - 2Ala - Thr + H]^+$	226.120	225.878	0.242	838.550	3.438
$[M - 2Ala - 2Thr + H]^+$	161.094	160.863	0.231	1205.688	39.945
$[M - 2H_2O - 3Ala - Thr + H]^+$	155.083	154.845	0.238	121.326	3.736

Table S6. Mass list of fragment ion peaks from proton adduct ion of linear ATAATA (precursor ion *m/z* 505.2) in Figure 4b.

Structure	Calculated *m/z*	Detected *m/z*	Δ*m/z*	Intensity	S/N
$[M + H]^+$	505.263	505.431	0.168	52635.032	250.701
$[M - H_2O + H]^+$	487.253	487.185	0.068	18550.935	78.679
$[M - 2H_2O + H]^+$	469.242	469.147	0.095	639.535	2.457
$[M - C_2H_4O + H]^+$	461.297	461.137	0.160	4923.727	18.918
$[M - Ala + H]^+$	434.226	434.078	0.148	652.201	2.752
$[M - 2C_2H_4O + H]^+$	417.211	417.122	0.089	3147.877	18.298
$[M - H_2O - Ala + H]^+$	416.216	416.067	0.149	18560.622	107.889
$[M - 2H_2O - Ala + H]^+$	398.205	398.033	0.172	3955.320	24.142
$[M - Ala - Thr + H]^+$	333.179	332.951	0.228	3613.944	15.244
$[M - H_2O - Ala - Thr + H]^+$	315.168	314.942	0.226	28657.720	123.399
$[M - 2H_2O - Ala - Thr + H]^+$	297.157	296.929	0.228	376.406	1.706
$[M - 2Ala - Thr + H]^+$	262.141	261.896	0.245	6403.957	59.014
$[M - H_2O - 2Ala - Thr + H]^+$	244.131	243.888	0.243	6190.802	58.565
$[M - 2H_2O - 2Ala - Thr + H]^+$	226.120	225.867	0.253	1577.833	16.091
$[M - 3Ala - Thr + H]^+$	191.104	190.846	0.258	2909.983	109.632
$[M - H_2O - 3Ala - Thr + H]^+$	173.094	172.842	0.252	851.192	40.493
$[M - 2H_2O - 3Ala - Thr + H]^+$	155.083	154.836	0.247	65.770	5.707

Table S7 Mass list of fragment ion peaks from proton adduct ion of linear AATAAT (precursor ion *m*/*z* 505.2) in Figure 4c.

Structure	Calculated *m*/*z*	Detected *m*/*z*	Δ*m*/*z*	Intensity	S/N
$[M + H]^+$	505.263	505.380	0.117	22474.433	128.523
$[M - H_2O + H]^+$	487.253	487.228	0.025	27868.107	140.454
$[M - 2H_2O + H]^+$	469.242	469.193	0.049	627.509	2.849
$[M - C_2H_4O + H]^+$	461.297	461.182	0.115	9314.797	42.292
$[M - Ala + H]^+$	434.226	434.108	0.118	398.667	1.663
$[M - H_2O - Thr + H]^+$	386.205	386.071	0.134	39514.474	118.258
$[M - 2H_2O - Thr + H]^+$	368.195	368.050	0.145	3220.805	9.919
$[M - 2Ala + H]^+$	363.189	363.038	0.151	6057.422	18.654
$[M - H_2O - Ala - Thr + H]^+$	315.168	314.986	0.182	19336.418	86.270
$[M - 2H_2O - Ala - Thr + H]^+$	297.157	296.973	0.184	6620.914	31.717
$[M - 2Ala - Thr + H]^+$	262.141	261.940	0.201	4706.729	38.853
$[M - H_2O - 2Ala - Thr + H]^+$	244.131	243.932	0.199	6070.477	65.872
$[M - 2H_2O - 2Ala - Thr + H]^+$	226.120	225.903	0.217	252.436	2.959
$[M - 3Ala - Thr + H]^+$	191.104	190.894	0.210	742.263	37.007
$[M - 2H_2O - 3Ala - Thr + H]^+$	155.083	154.862	0.221	55.949	4.526
$[M - H_2O - 2Ala - 2Thr + H]^+$	143.083	142.877	0.206	385.995	32.661
$[M - 4Ala - Thr + H]^+$	120.067	119.874	0.193	117.549	13.552

Table S8. Mass list of fragment ion peaks from sodium adduct ion of linear TAATAA (precursor ion *m/z* 527.2) in Figure 5a.

Structure	Calculated *m/z*	Detected *m/z*	Δ*m/z*	Intensity	S/N
$[M + Na]^+$	527.244	527.557	0.313	897.963	74.950
$[M - H_2O + Na]^+$	509.234	509.275	0.041	248.082	8.975
$[M - C_2H_4O + Na]^+$	483.218	483.255	0.037	6424.920	106.164
$[M - Ala + Na]^+$	456.207	456.216	0.009	10972.936	167.868
$[M - 2C_2H_4O + Na]^+$	439.192	439.191	0.001	253.551	3.781
$[M - H_2O - Ala + Na]^+$	438.197	438.153	0.044	189.374	2.824
$[M - C_2H_4O - Ala + Na]^+$	412.181	412.148	0.033	3619.039	57.020
$[M - 2Ala + Na]^+$	385.170	385.116	0.054	1246.812	38.801
$[M - 2C_2H_4O - Ala + Na]^+$	368.155	368.095	0.060	515.153	16.099
$[M - H_2O - 2Ala + Na]^+$	367.160	367.090	0.070	263.610	8.238
$[M - C_2H_4O - 2Ala + Na]^+$	341.144	341.075	0.069	859.797	33.129
$[M - 2C_2H_4O - 2Ala + Na]^+$	297.118	297.009	0.109	120.000	4.244
$[M - 2Ala - Thr + Na]^+$	284.122	284.011	0.111	2153.934	82.504
$[M - H_2O - 2Ala - Thr + Na]^+$	266.112	265.990	0.122	916.618	37.759
$[M - C_2H_4O - 2Ala - Thr + Na]^+$	240.096	239.977	0.119	186.752	8.319
$[M - 3Ala - Thr + Na]^+$	213.085	212.972	0.113	53.579	9.074
$[M - H_2O - 3Ala - Thr + Na]^+$	195.075	194.963	0.112	27.668	5.771
$[M - C_2H_4O - 3Ala - Thr + Na]^+$	169.059	168.935	0.124	12.188	3.163
$[M - 4Ala - Thr + Na]^+$	142.048	141.924	0.124	11.817	3.886

Table S9. Mass list of fragment ion peaks from sodium adduct ion of linear ATAATA (precursor ion *m*/*z* 527.2) in Figure 5b.

Structure	Calculated *m*/*z*	Detected *m*/*z*	Δ*m*/*z*	Intensity	S/N
$[M + Na]^+$	527.244	527.236	0.008	8823.960	30.179
$[M - H_2O + Na]^+$	509.234	509.228	0.006	21914.381	65.714
$[M - C_2H_4O + Na]^+$	483.218	483.195	0.023	57363.495	120.391
$[M - Ala + Na]^+$	456.207	456.163	0.044	133754.995	230.528
$[M - 2C_2H_4O + Na]^+$	439.192	439.139	0.053	6952.894	11.108
$[M - H_2O - Ala + Na]^+$	438.197	438.111	0.086	19857.853	31.726
$[M - C_2H_4O - Ala + Na]^+$	412.181	412.089	0.092	42093.306	68.767
$[M - 2C_2H_4O - Ala + Na]^+$	368.155	368.111	0.044	15017.398	22.016
$[M - Ala - Thr + Na]^+$	355.160	355.022	0.138	74118.856	117.350
$[M - H_2O - Ala - Thr + Na]^+$	337.149	336.997	0.152	5178.833	8.888
$[M - C_2H_4O - Ala - Thr + Na]^+$	311.133	310.982	0.151	7808.725	15.455
$[M - 2Ala - Thr + Na]^+$	284.122	283.956	0.166	11499.741	54.176
$[M - H_2O - 2Ala - Thr + Na]^+$	266.112	265.931	0.181	1436.903	8.902
$[M - C_2H_4O - 2Ala - Thr + Na]^+$	240.096	239.914	0.182	1156.667	11.878
$[M - 3Ala - Thr + Na]^+$	213.085	212.892	0.193	1377.341	31.997
$[M - H_2O - 3Ala - Thr + Na]^+$	195.075	194.871	0.204	429.580	14.158
$[M - C_2H_4O - 3Ala - Thr + Na]^+$	169.059	168.861	0.198	156.099	6.410
$[M - 3Ala - 2Thr + Na]^+$	112.038	111.858	0.180	136.168	6.305

Table S10. Mass list of fragment ion peaks from sodium adduct ion of linear AATAAT (precursor ion *m*/*z* 527.2) in Figure 5c.

Structure	Calculated *m*/*z*	Detected *m*/*z*	Δ*m*/*z*	Intensity	S/N
$[M + Na]^+$	527.244	527.231	0.013	8380.498	37.008
$[M - H_2O + Na]^+$	509.234	509.241	0.007	28524.742	108.550
$[M - C_2H_4O + Na]^+$	483.218	483.204	0.014	39681.646	94.831
$[M - C_2H_4O - H_2O + Na]^+$	465.208	465.233	0.025	70065.229	127.609
$[M - 2C_2H_4O + Na]^+$	439.192	439.141	0.051	2123.501	3.752
$[M - Thr + Na]^+$	426.197	426.163	0.034	90813.754	162.292
$[M - H_2O - Thr + Na]^+$	408.186	408.123	0.063	12962.267	22.066
$[M - H_2O - C_2H_4O - Ala + Na]^+$	394.170	394.323	0.153	2170.825	4.281
$[M - C_2H_4O - Thr + Na]^+$	382.170	382.108	0.062	9150.550	19.829
$[M - Ala - Thr + Na]^+$	355.160	355.091	0.069	11696.214	38.041
$[M - H_2O - Ala - Thr + Na]^+$	337.149	337.081	0.068	2202.404	8.053
$[M - C_2H_4O - H_2O - 2Ala + Na]^+$	323.133	323.070	0.063	410.347	1.950
$[M - C_2H_4O - Ala - Thr + Na]^+$	311.133	311.048	0.085	946.603	5.162
$[M - 2Ala - Thr + Na]^+$	284.122	284.028	0.094	6958.114	57.554
$[M - H_2O - 2Ala - Thr + Na]^+$	266.112	266.017	0.095	1454.662	15.533
$[M - C_2H_4O - 2Ala - Thr + Na]^+$	240.096	240.005	0.091	718.503	10.501
$[M - H_2O - C_2H_4O - 2Ala - Thr + Na]^+$	222.086	222.009	0.077	2192.465	47.212
$[M - 2Ala - 2Thr + Na]^+$	183.075	182.964	0.111	367.189	11.219

Table S11. Mass list of fragment ion peaks from sodium adduct ion of alanine deuterium-labeled cyclic AFGP core peptide (precursor ion *m*/*z* 521.2) in Figure 6a.

Structure	Calculated *m*/*z*	Detected *m*/*z*	Δ*m*/*z*	Intensity	S/N
$[M + Na]^+$	521.309	521.550	0.241	217724.074	394.357
$[M - H_2O + Na]^+$	503.299	503.331	0.032	40157.956	65.889
$[M - CO + Na]^+$	493.314	493.332	0.018	29234.257	44.446
$[M - 2H_2O + Na]^+$	485.288	485.257	0.031	1947.638	2.068
$[M - C_2H_4O + Na]^+$	477.283	477.279	0.004	74512.874	106.358
$[M - CO - H_2O + Na]^+$	475.304	475.221	0.083	5191.826	5.232
$[M - H_2O - C_2H_4O + Na]^+$	459.210	459.227	0.017	20931.909	22.132
$[M - CO - C_2H_4O + Na]^+$	449.288	449.231	0.057	16113.791	23.949
$[M - 2C_2H_4O + Na]^+$	433.257	433.180	0.077	16303.937	20.315
$[M - CO - H_2O - C_2H_4O + Na]^+$	431.277	431.147	0.130	2721.670	3.391
$[M - CO - Ala(d3) + Na]^+$	419.258	419.157	0.101	6142.480	11.327
$[M - CO - 2C_2H_4O + Na]^+$	405.262	405.133	0.129	3395.370	6.956
$[M - CO - Ala(d3) - H_2O + Na]^+$	401.248	401.104	0.144	1478.129	2.652
$[M - CO - Thr + Na]^+$	392.267	392.143	0.124	3520.629	8.325
$[M - CO - Ala(d3) - C_2H_4O + Na]^+$	375.232	375.094	0.138	4165.073	11.875
$[M - CO - 2Ala(d3) + Na]^+$	345.202	345.035	0.167	1500.066	6.159
$[M - CO - 2Ala(d3) - H_2O + Na]^+$	327.192	327.004	0.188	844.339	4.374
$[M - CO - Ala(d3) - Thr + Na]^+$	318.211	318.031	0.180	3365.928	20.656
$[M - CO - 2Ala(d3) - C_2H_4O + Na]^+$	301.176	300.995	0.181	865.216	6.209
$[M - CO - 2Ala(d3) - Thr + Na]^+$	244.155	243.956	0.199	3982.126	73.381
$[M - CO - 2Ala(d3) - Thr - H_2O + Na]^+$	226.144	225.912	0.232	389.693	8.155
$[M - CO - 2Ala(d3) - Thr - C_2H_4O + Na]^+$	200.128	199.912	0.216	401.647	12.308
$[M - CO - 3Ala(d3) - Thr + Na]^+$	170.099	169.900	0.199	260.553	9.739
$[M - CO - 2Ala(d3) - 2Thr + Na]^+$	143.107	142.909	0.198	194.908	9.214
$[M - CO - 4Ala(d3) - Thr + Na]^+$	96.043	95.849	0.194	77.773	3.251
$[M - CO - 3Ala(d3) - 2Thr + Na]^+$	69.051	68.885	0.166	72.004	2.563

Table S12. Mass list of fragment ion peaks from sodium adduct ion of cyclic TA(d3)A(d3)A(d3)A(d3)A(d3) (precursor ion *m/z* 494.2) in Figure 6b.

Structure	Calculated *m/z*	Detected *m/z*	Δ*m/z*	Intensity	S/N
$[M + Na]^+$	494.317	494.447	0.130	118746.087	234.153
$[M - H_2O + Na]^+$	476.307	476.292	0.015	7778.765	14.251
$[M - CO + Na]^+$	466.320	466.306	0.014	8070.321	14.297
$[M - C_2H_4O + Na]^+$	450.291	450.243	0.048	16464.206	28.391
$[M - CO - H_2O + Na]^+$	448.312	448.232	0.080	815.431	1.412
$[M - CO - C_2H_4O + Na]^+$	422.296	422.175	0.121	2421.129	14.014
[M - CO - Ala(d3) + Na]$^+$	392.267	392.105	0.162	2456.089	21.641
[M - CO - Ala(d3) - H_2O + Na]$^+$	374.256	374.067	0.189	449.084	4.762
[M - CO - Thr + Na]$^+$	365.275	365.085	0.190	637.535	7.578
[M - CO - Ala(d3) - C_2H_4O + Na]$^+$	348.240	348.027	0.213	1019.902	14.447
[M - CO - 2Ala(d3) + Na]$^+$	318.211	317.987	0.224	590.382	11.795
[M - CO - 2Ala(d3) - H_2O + Na]$^+$	300.200	299.934	0.266	134.763	3.027
[M - CO - Ala(d3) - Thr + Na]$^+$	291.219	290.954	0.265	613.674	15.982
[M - CO - 2Ala(d3) - C_2H_4O + Na]$^+$	274.184	273.916	0.268	189.957	5.672
[M - CO - 3Ala(d3) + Na]$^+$	244.155	243.870	0.285	232.131	9.496
[M - CO - 3Ala(d3) - H_2O + Na]$^+$	226.144	225.805	0.339	44.101	2.035
[M - CO - 2Ala(d3) - Thr + Na]$^+$	217.163	216.869	0.294	273.222	15.342
[M - CO - 3Ala(d3) - C_2H_4O + Na]$^+$	200.128	199.806	0.322	51.829	3.277
[M - CO - 4Ala(d3) + Na]$^+$	170.099	169.813	0.286	56.886	4.377
[M - CO - 4Ala(d3) - H_2O + Na]$^+$	152.088	151.812	0.276	57.299	4.730
[M - CO - 3Ala(d3) - Thr + Na]$^+$	143.107	142.825	0.282	129.199	11.036
[M - CO - 4Ala(d3) - C_2H_4O + Na]$^+$	126.072	125.780	0.292	24.540	2.221
[M - CO - 4Ala(d3) - Thr + Na]$^+$	69.051	68.855	0.196	48.530	3.794

Table S13. Mass list of fragment ion peaks from proton adduct ion of cyclic SAAAAA (precursor ion *m*/*z* 443.2) in Figure 7a.

Structure	Calculated *m*/*z*	Detected *m*/*z*	Δ*m*/*z*	Intensity	S/N
$[M + H]^+$	443.227	443.195	0.032	5827.465	70.434
$[M - H_2O + H]^+$	425.216	425.172	0.044	5418.869	57.745
$[M - CO + H]^+$	415.232	415.178	0.054	2609.327	25.532
$[M - Ala + H]^+$	372.189	372.162	0.027	9849.507	110.542
$[M - Ser + H]^+$	356.195	356.141	0.054	823.894	10.948
$[M - CO - Ala + H]^+$	344.195	344.203	0.008	407.351	5.668
$[M - CO - Ser + H]^+$	328.200	328.187	0.013	136.950	2.071
$[M - 2Ala + H]^+$	301.149	301.207	0.058	1365.673	24.315
$[M - Ala - Ser + H]^+$	285.157	285.212	0.055	1125.409	22.734
$[M - CO - 2Ala + H]^+$	273.157	273.212	0.055	136.896	3.174
$[M - CO - Ala - Ser + H]^+$	257.163	257.244	0.081	125.403	3.563
$[M - 3Ala + H]^+$	230.115	230.204	0.089	811.932	28.694
$[M - 2Ala - Ser + H]^+$	214.120	214.198	0.078	673.415	29.960
$[M - CO - 3Ala + H]^+$	202.120	202.185	0.065	97.692	5.003
$[M - 4Ala + H]^+$	159.078	158.953	0.125	147.414	15.943
$[M - 3Ala - Ser + H]^+$	143.083	142.774	0.309	122.412	16.198

Table S14. Mass list of fragment ion peaks from proton adduct ion of cyclic SAAAAA (precursor ion *m/z* 465.2) in Figure 7b.

Structure	Calculated *m/z*	Detected *m/z*	Δ*m/z*	Intensity	S/N
$[M + Na]^+$	465.208	465.211	0.003	54529.756	132.654
$[M - H_2O + Na]^+$	447.197	447.167	0.030	53503.896	123.678
$[M - CO + Na]^+$	437.213	437.183	0.030	37205.510	81.955
$[M - CH_2O + Na]^+$	435.197	435.170	0.027	12631.590	27.825
$[M - CO - H_2O + Na]^+$	419.202	419.159	0.043	3909.247	8.029
$[M - CO - CH_2O + Na]^+$	407.202	407.181	0.021	2257.988	4.486
$[M - Ala + Na]^+$	394.170	394.171	0.001	2574.415	14.496
$[M - CO - Ala + Na]^+$	366.176	366.217	0.041	14323.925	84.209
$[M - CO - Ser + Na]^+$	350.181	350.228	0.047	2130.040	13.765
$[M - CO - Ala - H_2O + Na]^+$	348.165	348.228	0.063	1903.438	12.301
$[M - CO - Ala - CH_2O + Na]^+$	336.165	336.242	0.077	1141.037	7.927
$[M - 2Ala + Na]^+$	323.130	323.237	0.107	917.168	6.513
$[M - 2Ala - H_2O + Na]^+$	305.123	305.245	0.122	789.734	7.428
$[M - CO - 2Ala + Na]^+$	295.138	295.271	0.133	2119.390	23.673
$[M - 2Ala - CH_2O + Na]^+$	293.123	293.230	0.107	168.855	1.886
$[M - CO - Ala - Ser + Na]^+$	279.144	279.288	0.144	2275.388	32.946
$[M - CO - 2Ala - H_2O + Na]^+$	277.128	277.264	0.136	389.147	5.635
$[M - CO - 2Ala - CH_2O + Na]^+$	265.128	265.272	0.144	313.809	5.226
$[M - 2Ala - Ser + Na]^+$	236.101	236.265	0.164	134.593	3.718
$[M - CO - 3Ala + Na]^+$	224.101	224.263	0.162	495.142	19.843
$[M - CO - 2Ala - Ser + Na]^+$	208.106	208.260	0.154	893.995	47.700
$[M - CO - 4Ala + Na]^+$	153.064	152.853	0.211	47.777	5.445
